# Dynamic Model of a Conjugate-Surface Flexure Hinge Considering Impacts between Cylinders

**DOI:** 10.3390/mi13060957

**Published:** 2022-06-16

**Authors:** Alessandro Cammarata, Pietro Davide Maddìo, Rosario Sinatra, Andrea Rossi, Nicola Pio Belfiore

**Affiliations:** 1 Department of Civil Engineering and Architecture, University of Catania, Viale Andrea Doria 6, 95123 Catania, Italy; pietro.maddio@unict.it (P.D.M.); rosario.sinatra@unict.it (R.S.); 2 Department of Industrial, Electronic and Mechanical Engineering, University of Roma Tre, Via Vito Volterra 62, 00154 Rome, Italy; andrea.rossi@uniroma3.it (A.R.); nicolapio.belfiore@uniroma3.it (N.P.B.)

**Keywords:** multibody systems, CSFH, event-driven scheme, non-smooth contact, LN-model, Moreau time-stepping scheme

## Abstract

A dynamic model of a Conjugate-Surface Flexure Hinge (CSFH) has been proposed as a component for MEMS/NEMS Technology-based devices with lumped compliance. However, impacts between the conjugate surfaces have not been studied yet and, therefore, this paper attempts to fill this gap by proposing a detailed multibody system (MBS) model that includes not only rigid-body dynamics but also elastic forces, friction, and impacts. Two models based on the Lankarani-Nikravesh constitutive law are first recalled and a new model based on the contact of cylinders is proposed. All three models are complemented by the friction model proposed by Ambrosìo. Then, the non-smooth Moreau time-stepping scheme with Coulomb friction is described. The four models are compared in different scenarios and the results confirm that the proposed model outcomes comply with the most reliable models.

## 1. Introduction

During the last decades, the development of both MEMS (micro electro-mechanical systems) and NEMS (nano electro-mechanical systems) technology-based devices encountered several technological issues [1]. As far as the mechanical structure is concerned, the micromachining methods and the available materials inevitably restricted the mobility of most micro/nanosystems mobility to a plane motion with a few degrees of freedom (DoF), when not even down to a single DoF only.

The appearance of flexure hinges, together with lumped compliant structure, disclosed new ways to design. As a consequence, several new devices, obtained by means of micromachining, were proposed in the literature. For example, biosensing acoustic wave based devices [2], CMOS-MEMS resonators [3], microgrippers [4], drug delivery micropumps [5], surgery [6], micromirror platforms [7], and more generally actuators [8,9] and sensors [10].

A peculiar hinge, called Conjugate-Surface Flexure Hinge (CSFH), has been successfully proposed as a component for MEMS/NEMS Technology-based devices with lumped compliance [11,12] and the next section will be dedicated to some important details on CSFHs.

Although several aspects of CSFH equipped microsystems have been already studied, such as, adaptability to precision mechanism [13], kinetostatics [14], operational in aqueous environment [15], vibrations [16] and technological issues [17], the dynamical behavior of a CSFH still remains unexplored. For example, the impacts and their consequences on dynamics have not been studied yet. Contact is an inherent feature of the CSFH but can yield wear [18].

The contact in mechanical joints with clearance has been largely discussed in the literature [19]. Since the CSFH conjugate surfaces can be described as a journal-bearing, here, we refer only to this class of joint. The two most commonly used approaches to describe the phenomenon of impact see a continuous regularized approach [20,21,22] versus a non-smooth approach [23,24]. The regularized approach derives the contact laws using geometric and material parameters of the contacting surfaces, the non-smooth approach does not require impact laws and the impact is instantaneous. Although both approaches are valid, the dynamics of impact can be quite different, especially in the presence of external forces capable of amplifying the small differences coming from different contact dynamic responses.

For this reason, we will attempt to fill this gap by proposing a detailed multibody system (MBS) model that includes rigid-body dynamics, elastic forces, friction, and impacts. This complex mathematical tool must be flexible and provide reliable results. Motivated by this reason, starting from models widely employed in multibody systems with impacts and experimentally validated such as those reported in [20,21,22], we propose a novel model in which the generalized stiffness obtained considering the impact of two cylinders and not two spheres. Results will demonstrate that our model is consistent with other experimentally verified models.

In Section 3, the circular beam flexure hinge is described and the generalized elastic forces to include in the dynamic model are obtained. Section 4 introduces continuous impact models based on regularized approaches. Hertz contact theory is first recalled, then the Lankarani-Nikravesh model and its modified version with a non-constant generalized contact stiffness are described. Starting from the classic Lankarani-Nikravesh constitutive law, a novel method based on the contact of two cylindrical surfaces is presented. All three continuous impact models are complemented with the friction model proposed by Ambrosìo. In Section 5, the non-smooth Moreau time-stepping scheme with Coulomb friction is described. Section 6 compares the methods of the previous sections considering different impact scenarios. First, a central and an asymmetrical impact are described. Then, a complete CSFH dynamics is simulated. Section 7 deals with the influence of model parameters on system dynamics. Finally, Section 8 and Section 9 summarize the final comments, future developments and conclusions.

## 2. Motivations and Contributions

The main motivations that guided the study are listed below:

1. absence of a complete dynamic model of a CSFH,
2. create a specific impact model for CSFH that is a valid alternative to those most commonly used in the literature,
3. provide some guidelines for proper CSFH modeling and design.

These motivations have led to the following main contributions:

1. developing a detailed multibody model of a CSFH including rigid-body dynamics, elastic forces, friction, and impacts,
2. developing a novel event-driven model considering impacts between cylinders,
3. conducting a parametric analysis to understand the influence of each parameter on the CSFH dynamics.

## 3. Flexure Description

The Conjugate-Surface Flexure Hinge (CSFH) is composed of two parts: a flexure beam connecting two bodies and a conjugate-surface area where the two bodies can either slide or collide. The curved beam leads to nonlinear motion characteristics [25]. While the kineto-static analysis of CSFH has been already described in detail [26], this paragraph recalls some relevant outlines that will be useful for the sake of the present investigation.

### 3.1. Kinematics

**Figure 1:**
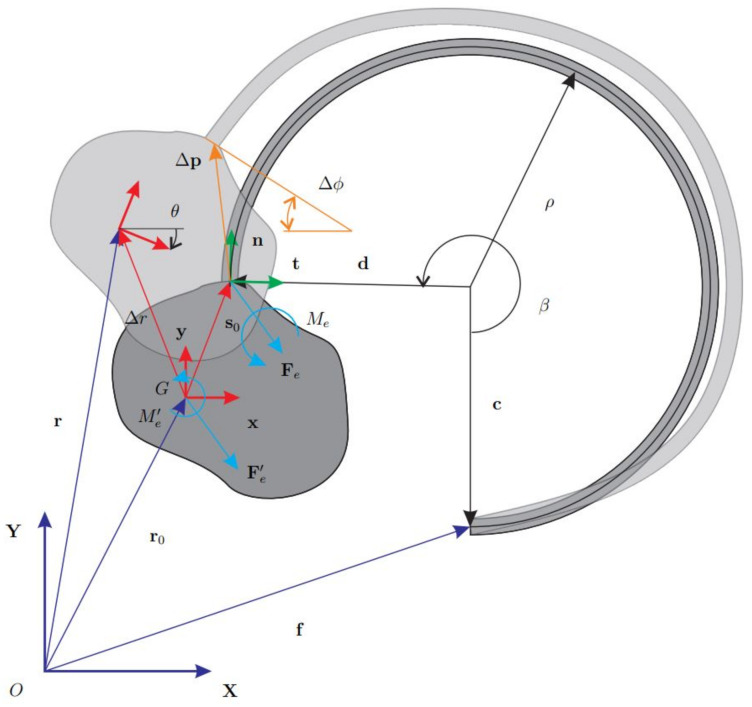
Layout of a CSFH.

The basic layout illustrated in Figure 1 will be the reference model for the dynamic analysis. Accordingly, the flexure hinge is connected to a rigid body, where both undeformed and deformed configurations have been displayed.

Kinetostatic analysis will be herein introduced through the vector

(1) $$r_{0}=f-c+d-s_{0}$$

that defines the mass-center position of the rigid body in the undeformed configuration, where:

- f stands for the position of the beam root,
- c and d, respectively, denote the vectors from the center of the circular hinge to the endpoints of the curved beam,
- $s_{0}$ is the vector connecting the mass-center to the body-hinge attachment point in the undeformed configuration.

Being ρ and β the flexure radius and opening angle, respectively, the vector h=d−c, can be expressed as

(2) $$h=\rho \left[\begin{array}{c} c(\beta -\frac{\pi }{2})\\ 1+s(\beta -\frac{\pi }{2}) \end{array}\right]$$

where the compact notation s≡sin and c≡cos has been employed. Vector $s_{0}$ connects two rigid-body points and can be expressed considering the rotation matrix A of the body in its undeformed configuration and the vector $\bar{s}$ relative to the body-frame (x,y), i.e.

(3) $$s_{0}=A\left(\theta_{0}\right)\bar{s}\Rightarrow \left[\begin{array}{c} s_{0x}\\ s_{0y} \end{array}\right]=\left[\begin{array}{cc} c(\theta_{0}) & -s(\theta_{0})\\ s(\theta_{0}) & c(\theta_{0}) \end{array}\right]\left[\begin{array}{c} {\bar{s}}_{x}\\ {\bar{s}}_{y} \end{array}\right]$$

where θ is the angle between the axes x and X. If $\theta_{0}=0$, it follows that $s_{0}\equiv \bar{s}$. The expression of $r_{0}$ becomes

(4) $$\left[\begin{array}{c} r_{0x}\\ r_{0y} \end{array}\right]=\left[\begin{array}{c} f_{x}+\rho c\left(\beta -\frac{\pi }{2}\right)-{\bar{s}}_{x}\\ f_{y}+\rho +\rho s\left(\beta -\frac{\pi }{2}\right)-{\bar{s}}_{y} \end{array}\right]$$

being $f_{x}$ and $f_{y}$ the components of f in the reference frame.

Consider the deformed configuration and taking $p=r+A\bar{s}$ as the positioning vector of the body-hinge attachment point, the displacement Δp is simply expressed as

(5) $$\Delta p=p-p_{0}\equiv r+A\bar{s}-r_{0}-s_{0}\equiv \Delta r+\left(A-1\right)\bar{s}$$

in which Δr is the mass-center displacement. As it can be observed in Figure 1, the rotation Δϕ of the attachment section is equal to θ. The standard stiffness model for this case [26] has been obtained by considering the local frame (n,t), respectively, composed of the vectors normal and tangent to the attachment cross-section. This frame moves with the body and has a constant orientation with respect to the body-frame (x,y). The rotation matrix R expressing this constant orientation has the following expression

(6) $$R=\left[\begin{array}{cc} c(\psi ) & -s(\psi )\\ s(\psi ) & c(\psi ) \end{array}\right],\;\psi =2\pi -\beta$$

Composing R and A is possible to pass from the local frame (n,t) to the global frame (X,Y). In the following, the steps necessary to find the generalized elastic force vector are detailed.

### 3.2. Elastic Force

The above-mentioned stiffness model can be summarized through the following expression:

(7) $$\left[\begin{array}{c} \Delta \bar{p}\\ \Delta \phi \end{array}\right]=\hat{C}\left[\begin{array}{c} {\hat{F}}_{e}\\ M_{e} \end{array}\right]$$

where $\hat{C}$ is the compliance matrix [26]. The force vector ${\hat{F}}_{e}$ contains the normal and tangential forces expressed in the frame (n,t) and applied at the attachment section while $M_{e}$ is the moment. These components can be gathered into the generalized vector $\hat{w}={\left[{\hat{F}}_{e}^{T},M_{e}\right]}^{T}$.

Given the generic configuration of the rigid body expressed through the three-elements array $q={\left[r^{T},\theta \right]}^{T}$ the following procedure will be used.

1. find Δp using Equation (5),
2. find the vector $w_{e}={\left[F_{e}^{T},M_{e}\right]}^{T}$ defined in (X,Y):
  (8) $$w_{e}=K\Delta p,\;K=AR\hat{K}R^{T}A^{T}$$
  where K is the stiffness matrix expressed in the reference frame (X,Y) and $\hat{K}\equiv {\hat{C}}^{-1}$ is the local stiffness matrix in (n,t),
3. transport the vector $w_{e}$ to the mass-centre point *G* to find the generalized force vector $Q_{e}={\left[{\hat{F^{\prime }}}_{e}^{T},M_{e}^{\prime }\right]}^{T}$, defined as
  (9) $$Q_{e}=T_{e}w_{e}\equiv \left[\begin{array}{cc} 1 & 0\\ s^{T}\bar{1} & 1 \end{array}\right]\left[\begin{array}{c} F_{e}\\ M_{e} \end{array}\right],\;\bar{1}=\left[\begin{array}{cc} 0 & 1\\ -1 & 0 \end{array}\right]$$
  where $T_{e}$ is the 3×3 rigid-body transformation matrix needed to transport $w_{e}$ and $\bar{1}$ is necessary to consider the cross-product in the planar case [27].

## 4. Event-Driven Scheme with Regularized Approach

We first describe an event-driven scheme with a contact detection algorithm. These schemes integrate the equations of motion until a slip-stick transition or an impact is detected.

### 4.1. Contact Kinematics

**Figure 2:**
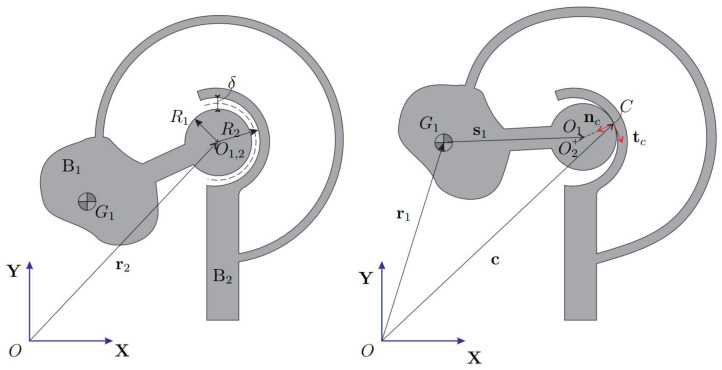
Undeformed CSFH (**left**). Deformed CSFH with impact (**right**).

The CSFH limits the hinge deformation by introducing two conjugate surfaces where the two bodies can collide. This solution has positive effects on motion accuracy and improves resistance to yielding as well. Figure 2 shows the undeformed and deformed CSFH. The conjugate surfaces, represented through two circles of radii $R_{1}$ and $R_{2}$, are separated by a radial clearance $\delta =R_{2}-R_{1}$ in the undeformed configuration. The local vector ${\bar{s}}_{1}$ denotes the position of the center $O_{1}$ with respect to the body frame. During motion, the hinge deforms and the bodies collide at one point *C*. Observing the Figure 2, the following closure equation can be written

(10) $$c_{1}=c_{2}\Rightarrow r_{1}+A{\bar{s}}_{1}-R_{1}n_{c}=r_{2}-R_{2}n_{c}$$

where point *C* is thought to belong to the two bodies. Here, the second body is fixed for convenience. Equation (10) provides the unit vector $n_{c}$ normal to the conjugate surfaces at point *C*, i.e.,

(11) $$n_{C}=-\frac{e}{∥e∥}\;e=r_{1}+A{\bar{s}}_{1}-r_{2}$$

where e is the eccentricity vector expressing the position of $O_{1}$ with respect to $O_{2}$. The tangent unit vector $t_{c}$ is calculated rotating $n_{c}$$90^{\circ }$ counter-clockwise. Time-differentiating the expression of $c_{1}$, the velocity ${\dot{c}}_{1}$ is obtained as

(12) $${\dot{c}}_{1}={\dot{r}}_{1}+\Omega \left(s_{1}-R_{1}n_{c}\right)$$

being Ω the angular-velocity matrix of the first body. The expression of ${\dot{c}}_{1}$ is required to calculate the tangent velocity, that is the component of ${\dot{c}}_{1}$ along $t_{c}$.

### 4.2. Contact Model with Friction

In this subsection, different Hertzian contact models are first described. Then, the static friction force model of Ambrósio is recalled.

#### 4.2.1. Impact Models

The regularized approach to contact starts from the work by Hertz on the theory of elasticity [28,29]. Hertz introduced a non-linear law between the normal contact force $F_{N}$ and the indentation ϱ, i.e.,

(13) $$F_{N}=K\varrho^{n}$$

where *K* is the generalized stiffness parameter and *n* is the nonlinear exponent factor. The stiffness *K* is evaluated following Hertz contact theory. Considering two spheres, the following expression is obtained

(14) $$K=\frac{4}{3(\sigma_{1}+\sigma_{2})}\sqrt{\frac{R_{1}R_{2}}{R_{2}+R_{1}}}$$

where $\sigma_{i}$, i=1,2, are material parameters expressed in terms of Young’s modulus *E* and Poisson’s ratio η, defined by

(15) $$\sigma_{i}=\frac{1-\nu_{i}^{2}}{E_{i}},\;i=1,2$$

Considering two cylinders of length *L* with parallel axes, we have a contact on a rectangular area. In this case, Hertz proposed to use

(16) $$K=\frac{\pi }{4}E^{*}$$

where $E^{*}$ is an equivalent Elastic modulus, defined as

(17) $$\frac{1}{E^{*}}=\frac{1-\nu_{1}^{2}}{E_{1}}+\frac{1-\nu_{2}^{2}}{E_{2}}$$

The ESDU-78035 Tribology Series [30] proposed to use the following implicit law instead

(18) $$\varrho =F_{N}\left(\frac{\sigma_{1}+\sigma_{2}}{L}\right)\left[\ln\left(\frac{4L(R_{i}-R_{j})}{F_{N}\left(\sigma_{1}+\sigma_{2}\right)}\right)+1\right]$$

In this case, the stiffness *K* is not constant and can be obtained numerically from the derivative of the force-indentation curve.

Hertz law does not include energy dissipation due to internal damping. Therefore, following the work by Kelvin and Voigt, [31], different viscoelastic models have been proposed in the literature, [32]. The generic viscoelastic model has the following form

(19) $$F_{N}=K\varrho^{n}+D\varrho^{m}\dot{\varrho }$$

where *D* is the damping coefficient representing the dissipative term proportional to the relative normal contact velocity $\dot{\varrho }$ and *m* is a non-linear coefficient making the dissipation dependent on the indentation. The coefficient *m* can be empirical or based on dissipation models.

Hunt and Crossley [33] proposed a dissipative contact force model adding a non-linear viscoelastic term to Hertz law, thus coming to the following force-penetration law

(20) $$F_{N}=K\varrho^{n}+D\dot{\varrho }$$

Hunt and Crossley law can be written in terms of the coefficient of restitution $c_{r}$, i.e.

(21) $$F_{N}=K\varrho^{n}\left[1+\frac{3(1-c_{r})}{2}\frac{\dot{\varrho }}{{\dot{\varrho }}^{\left(-\right)}}\right]$$

being ${\dot{\varrho }}^{\left(-\right)}$ the initial contact velocity. Following Hunt and Crossley law, Lankarani-Nikravesh proposed an impact model to be applied in multibody systems [20]. The normal force is written as

(22) $$F_{N}=K\varrho^{n}\left[1+\frac{3(1-c_{r}^{2})}{4}\frac{\dot{\varrho }}{{\dot{\varrho }}^{\left(-\right)}}\right]$$

Since the Lankarani-Nikravesh model applies to stiff materials with the coefficient of restitution greater than 0.9, Flores et al. proposed an equivalent model for soft materials [34], i.e.,

(23) $$F_{N}=K\varrho^{n}\left[1+\frac{8(1-c_{r})}{5c_{r}}\frac{\dot{\varrho }}{{\dot{\varrho }}^{\left(-\right)}}\right]$$

In this case, the impact law considers different energy dissipation between compression and restitution of the contact phases.

In [22] the authors proposed to use a constitutive law of type

(24) $$F_{N}=K\varrho^{n}+D_{LN}\frac{K_{ESDU}}{K}\dot{\varrho }$$

to describe the journal-bearing contact. While *K* follows from Equation (14), $K_{ESDU}$ is derived from the ESDU law in Equation (18). Finally, $D_{LN}$ is the damping obtained using the Lankarani-Nikravesh (LN) model (22), i.e.,

(25) $$D_{LN}=\frac{3K(1-c_{r}^{2})}{4{\dot{\varrho }}^{\left(-\right)}}$$

This model hereafter referred to as the LNA-ESDU, has been experimentally verified for a slider-crank mechanism involving contact events at low or moderate impact velocities. Compared to the classic LN model, LNA-ESDU provides lower impact forces and is capable to accurately reproduce the experimental results probably because the actual impact forces are distributed on an wider area due to the plasticity deformation of the bristles.

Considering this literature, in this work we broaden the classic LN model of Equation (22) to also include the generalized stiffness obtained by the contact of two cylinders as in Equation (16).

In the numerical part, this model will be compared to the other LNA-based models for validation.

#### 4.2.2. Friction Model

Static friction force models start with the work of Coulomb [35]. Modified Coulomb’s laws such as those proposed by Threlfall [36] or Bo and Pavelescu [37] included the Stribeck effect, i.e., the transition from static to dynamic friction.

In most of these static models, the friction force can have a discontinuity at zero velocity. To solve this issue, Karnopp [38], Leine et al. [39], Bengisu and Akay [40] proposed static models with finite slope at zero velocity.

Nevertheless, in stick conditions, the relative tangent velocity should be zero but it does not occur due to numerical issues. Rather, it maintains close to zero and switches its sign with high frequency introducing numerical instability in the system’s response. To prevent this unwanted behavior, the Ambrósio static friction model put the friction force to zero for low velocities [41]. The friction force becomes

(26) $$F_{T}=\left\{\begin{array}{cc} 0 & |v_{T}|\leq v_{0}\\ -\frac{|v_{T}|-v_{0}}{v_{1}-v_{0}}f_{d}F_{N}\mathrm{sgn}\left(v_{T}\right) & v_{0}<\left|v_{T}\right|<v_{1}\\ -f_{d}F_{N}\mathrm{sgn}\left(v_{T}\right) & |v_{T}|\geq v_{1} \end{array}\right.$$

in which $f_{d}$ is the kinetic coefficient of friction, $v_{0}$ is the stiction velocity, $v_{1}$ is the slip velocity [38], and $v_{T}=\left(t_{c}^{T}v\right)t_{c}$ and $v_{T}$ are the relative tangential velocity and its module, respectively. In this case, $v\equiv {\dot{c}}_{1}$ reported in Equation (12).

For its stability, the Ambrósio static friction model is often used in multibody systems with frictional joints. Many other friction models could be coupled to the impact law and several empirical models have been presented in the literature. We refer to [42,43] for further details.

### 4.3. Equations of Motion

During the motion, the first body $B_{1}$ is subject to different generalized forces: the generalized elastic force $Q_{e}$, the gravitational force $Q_{g}$, and the generalized contact force $Q_{c}$. The latter is present only if the contact is triggered. Therefore, the undamped equations of motion are

(27) $$M\ddot{q}=Q$$

where the generalized force Q changes its expression according to the contact condition ∥e∥≥δ, i.e.,

(28a) $$\begin{array}{ccc} Q & = & Q_{e}+Q_{g},\;\mathrm{free}\mathrm{motion} \end{array}$$

(28b) $$\begin{array}{ccc} Q & = & Q_{e}+Q_{g}+Q_{c},\;\mathrm{contact} \end{array}$$

The generalized gravitational force is given by $Q_{g}={\left[m_{1}g^{T},0\right]}^{T}$, being $m_{1}$ the mass of $B_{1}$ and g the gravity acceleration vector.

The contact is triggered when ∥e∥≥δ. If this condition is met, a contact force $F_{c}$ is generated in the contact area. This force has two components along $n_{c}$ and $t_{c}$, respectively, i.e.,

(29) $$F_{c}=F_{N}n_{c}+F_{T}t_{c}$$

where $F_{N}$ is the normal force and $F_{T}$ is the friction force calculated following contact and friction models presented in the previous subsection. When carried to the mass center position $G_{1}$, the contact force $F_{c}$ yields a moment $M_{c}$, thus the 3-dimensional generalized contact force $Q_{c}$ can be expressed as

(30) $$Q_{c}\equiv \left[\begin{array}{c} F_{c}\\ M_{c} \end{array}\right]=\left[\begin{array}{c} 1\\ s_{1}^{T}\bar{1} \end{array}\right]F_{c}$$

**Figure 3:**
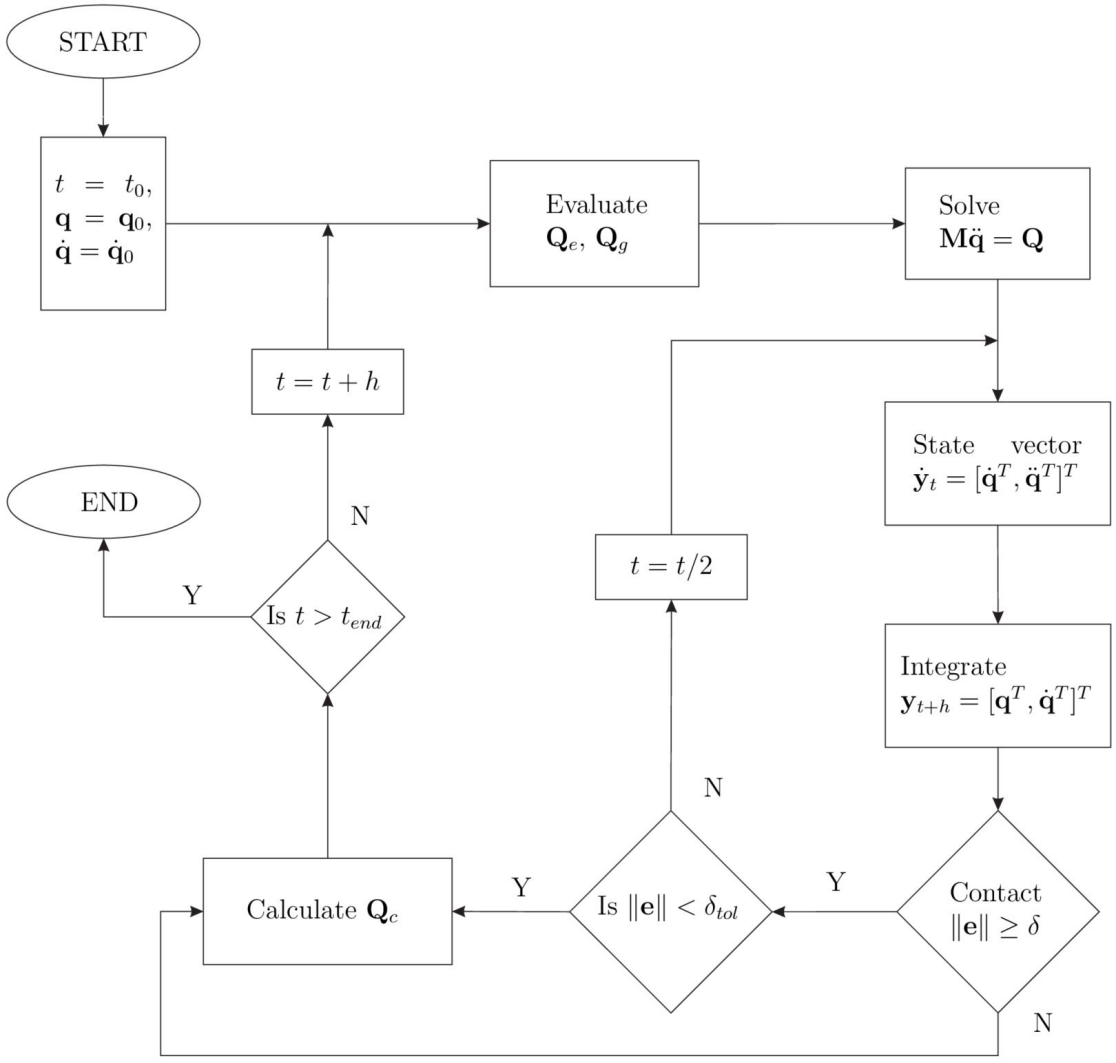
Flowchart of the event-driven scheme.

In Figure 3 the flowchart of procedure for dynamic analysis including impact is shown, [21,32]. When an impact condition is fulfilled, trying to get closer to the instant of the impact, a maximum tolerance $\delta_{tol}$ activates the bisection of the time interval. The generalized force applied to the system changes according to Equation (28).

## 5. Time-Stepping Method: Moreau’s Scheme

In contrast to the previous method, time-stepping methods do not require the detection of impact points and the consequent change of state. Rather, a discrete state is determined and held over the entire time step. These methods are less accurate than the previous event-driven schemes but are more robust and easy-to-implement. Here, Moreau’s scheme with the midpoint rule has been implemented [23,24]. Readers interested to time-stepping methods are referred to specialized bibliography [44,45,46]. In Moreau’s scheme, the equations of dynamics (27) are written as an index-2 DAE (differential algebraic equation) system, i.e.,

(31) $$\left\{\begin{array}{c} M\dot{u}-Q_{e}-Q_{g}-D^{T}\lambda =0\\ u=\dot{q}\\ -\gamma \equiv -Du\in N_{A}\left(\lambda \right) \end{array}\right.\;.$$

where u is the generalized velocity vector, D is the Jacobian matrix, λ is the vector of Lagrangian multipliers, γ is the time-derivative of the unilateral constraints, and $N_{A}$ is the cone of inclusion defined on the set *A*. The third equation is a set-valued law on velocity level that can be activated or not depending on a geometric gap function able to trigger the unilateral contact. Here, the gap function *g* can be defined as

(32) $$g\left(q\right)=\delta -\sqrt{e^{T}e}$$

where e has been defined in Equation (11). If g(q)≤0 the unilateral contact is active and the normal force modulus is

(33) $$F_{N}=\lambda_{1},\;\lambda_{1}\in R_{0}^{+}\equiv A_{1}$$

Now, the normal contact is defined at position level and not at velocity level, as required by Moreau’s time-stepping scheme. The time-derivative of the unilateral constraint g(q)=0 yields

(34) $$\gamma_{1}=\left[\begin{array}{cc} n_{c}^{T} & n_{c}^{T}\bar{1}s_{1} \end{array}\right]\dot{q}$$

where $\gamma_{1}=dg\left(q\right)/dt$.

The same gap function *g* activates also the tangential or friction force $F_{T}$. Once the impact has been triggered, the friction force can be either in impressed or constrained mode. In impressed mode there is slip between the surfaces in contact and $F_{T}$ is defined as

(35) $$F_{T}=-f_{d}\lambda_{1}\frac{v_{T}}{v_{T}}$$

Notice that here $F_{T}$ is not following the smooth transition provided by Equation (26).

In constrained mode there is stiction and $F_{T}$ belongs to the interval

(36) $$F_{T}\in \left[-f_{a}\lambda_{1},+f_{a}\lambda_{1}\right]$$

The same expression can be written in terms of the law of inclusion using the relative tangential velocity module $v_{T}$, i.e.,

(37) $$-v_{T}\in N_{A_{2}}\left(\lambda_{2}\right),\;A_{2}=\{\lambda_{2}\in R\;|\;|\lambda_{2}\left|\leq f_{a}\lambda_{1}\right\}$$

The latter expression allows defining a kinematic set-valued law in which $\gamma_{2}\equiv v_{T}$, i.e.,

(38) $$\gamma_{2}=\left[\begin{array}{cc} t_{c}^{T} & t_{c}^{T}\bar{1}\left(s_{1}-R_{1}n_{c}\right) \end{array}\right]\dot{q}\Rightarrow \gamma_{2}=\left[\begin{array}{cc} t_{c}^{T} & t_{c}^{T}\bar{1}s_{1} \end{array}\right]\dot{q}$$

Combining the Equations (34) and (38), we write

(39) $$\gamma \equiv Du,\;D=\left[\begin{array}{cc} n_{c}^{T} & n_{c}^{T}\bar{1}s_{1}\\ t_{c}^{T} & t_{c}^{T}\bar{1}s_{1} \end{array}\right]$$

in which D is the 2×3 Jacobian matrix of system (31).

## 6. Numerical Simulations

**Figure 4:**
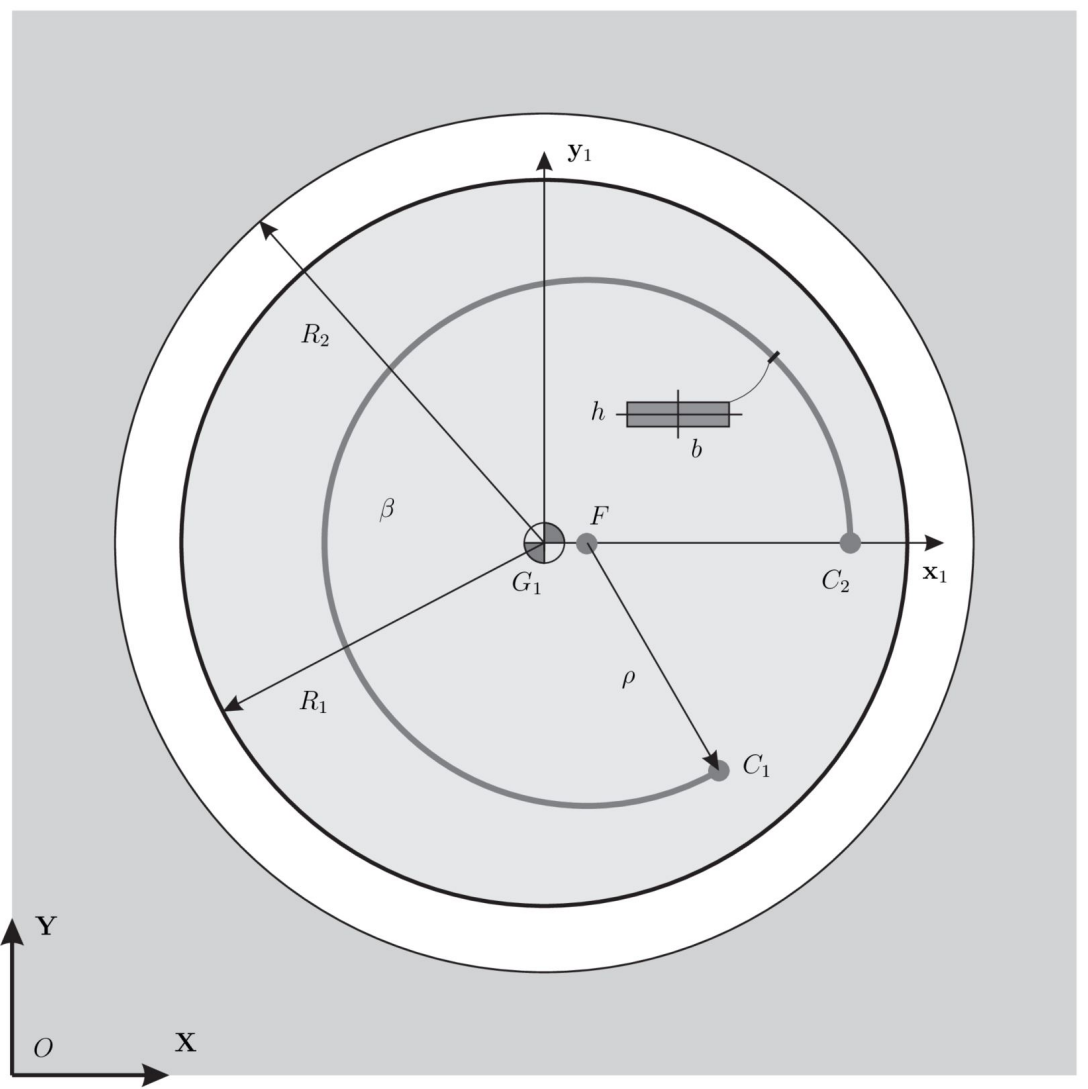
Layout of the CSFH used in the numerical simulations. Initial undeformed configuration.

The proposed formulation has been tested considering the CSFH displayed in Figure 4. Without any loss of meaning, we suppose that the mass centers $G_{1}$ and $G_{2}$ are, respectively, located at the geometric centers $O_{1}$ and $O_{2}$ of the conjugate cylindrical surfaces. Geometrical and structural parameters are reported in Table 1. Even if impact and friction follow different models, some parameters such as the restitution coefficient $c_{r}$, and the dynamic friction coefficient $f_{d}$ are common to both event-driven and time-stepping models.

In the following, four models will be compared:

- the classic Lankarani-Nikravesh impact model with generalized stiffness *K* as in Equation (14) + the modified Ambrósio friction model (LNA *K* spheres),
- the novel Lankarani-Nikravesh impact model with generalized stiffness *K* as in Equation (16) + the modified Ambrósio friction model (LNA *K* cylinders) proposed in this paper,
- the Lankarani-Nikravesh/ESDU impact model [22] + the modified Ambrósio friction model (LNA-ESDU),
- the Moreau time-stepping scheme.

As recalled, the first model is the classic LN impact model described in [20]. The second one is a modified version proposed in this paper that takes into account the contact of two cylinders modifying the generalized stiffness *K* through Equation (16). The LNA-ESDU has been validated experimentally for a journal-bearing contact with clearance of a slider-crank mechanism in [22]. Finally, the fourth model is the Moreau time-stepping scheme. It is noteworthy that the first three models are continuous models with event-driven schemes while the fourth is a time-stepping method. This article offers a first numerical comparison to understand if the proposed method is valid and comparable with methods widely accepted by the scientific community. The choice of using three models for comparison is linked to their importance in the multibody field. In fact, the LN model and the Moreau model are the most popular models, each for its own category. The ESDU model is more recent and does not have the same notoriety as the previous methods; however, it proved to be very reliable from an experimental point of view.

**Figure 5:**
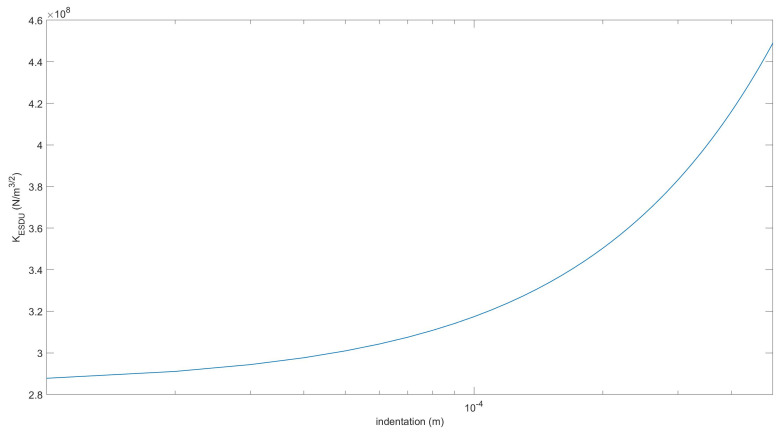
Generalized stiffness evaluated using the Lankarani–Nikravesh/ESDU impact model [22].

The parameters used in the three continuous models are reported in Table 2 and Table 3. It can be observed that the generalized stiffness parameter used for the classic LN impact model, i.e., considering the contact of two spheres, is one order of magnitude stiffer than that employed in our modified version in which the contact of two cylinders is considered. The same feature can be observed in Figure 5 for the LNA-ESDU where the generalized stiffness parameter is not constant but grows with the indentation [22]. The dynamic simulation has been performed using the explicit Runge-Kutta 4th-order method. The initial time step has been set to $h_{0}=$ 2 × 10${}^{-4}$ (s) while the tolerance of the event-driven scheme is $\delta_{tol}$ = 1 × 10${}^{-4}$ (m).

The Moreau model employs the parameters reported in Table 1. The time-stepping method is based on the midpoint rule with a fixed time step $h_{0}=$ 1× 10${}^{-5}$ (s).

### 6.1. Central Impact

**Figure 6:**
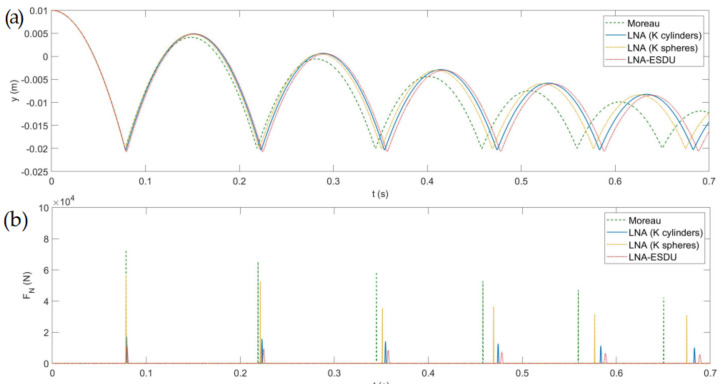
Model comparison considering a central impact: (**a**) *y*-coordinate of $G_{1}$, (**b**) impact force $F_{N}$.

First, we considered the simplest case of a central impact on the two conjugate surfaces. In this particular scenario, the friction force is zero making it possible to evaluate the differences between the four models in the impact process only. The initial state of body 1 is $q_{0}={\left[0,0.01,0\right]}^{T}$. The results of the simulation are displayed in Figure 6 where the vertical displacement of body 1 and the impact force are plotted. It can be observed that, after free-flying, body 1 impacts at the same instant for the four models, and the bounce height gradually decreases in time due to the dissipative effects. Since the Moreau model is non-smooth, its contact is impulsive and the impact force is the highest among the four models. The three smooth continuous models have the common feature that the impact is spread over a finite, albeit very small, time interval, therefore reducing the contact force peaks. Moreover, it can be observed that the dissipated energy is lower than in the Moreau model and that the sequence of impacts is dilated. Comparing the three continuous LNA-based models, we realize that the LNA (*K* cylinders) is the most rigid model with lower indentation. The remaining two models have similar characteristics with lower contact forces and greater penetration depths. From this simple experiment, we understand that although some common parameters employed in the models are the same, the comparison of the results shows dynamics that gradually become different. The discrepancies between regularized smooth LNA-based methods and non-smooth methods should not be surprising as they are inherent in different formulations. The contact in the LNA-based models is divided into two phases, the impact and the restitution phase while in the Moreau scheme the contact is non-smooth and impulsive and the rebound height is predominantly influenced by the restitution coefficient, here considered equal for all models. The discrepancies between the models are reduced when the materials are sufficiently rigid since the contact phase is reduced, tending to the limit case of impulsive contact.

**Figure 7:**
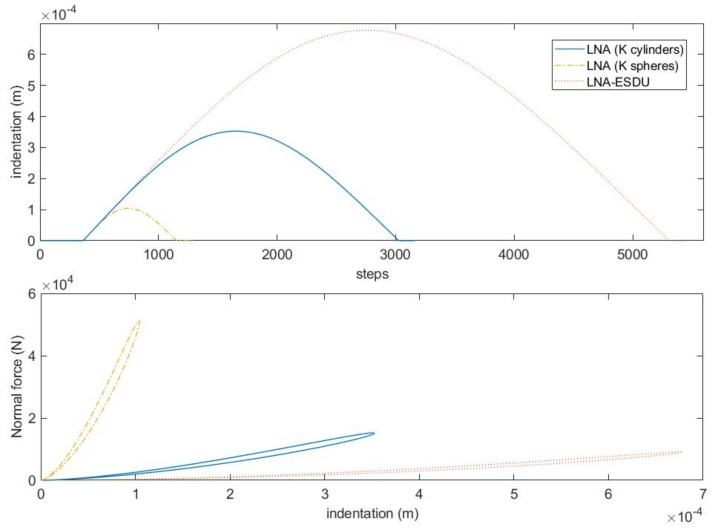
Model comparison considering an asymmetrical impact: from the top to the bottom: indentation vs. number of steps; hysteresis cycles.

To better understand the differences between the three continuous models, let’s analyze the first contact. Figure 7 shows the indentation curves and the hysteresis cycles. It can be observed that the three models have maximum indentation decreasing with the generalized stiffness. The LNA-ESDU is the model with a wider curve and with a flatter hysteresis cycle while the classic LNA model is the most rigid one. Finally, the LNA model with *K* calculated for two cylinders in contact is placed between the two. It should also be observed that the longer duration of the contact for the LNA-ESDU entails a greater computational burden that gradually decreases up to the classic LNA model.

### 6.2. Asymmetrical Impact

Let us now consider an asymmetrical collision where the friction force also comes into play. In this second scenario the initial state vector is $q_{0}={\left[0.01,0.01,0\right]}^{T}$.

**Figure 8:**
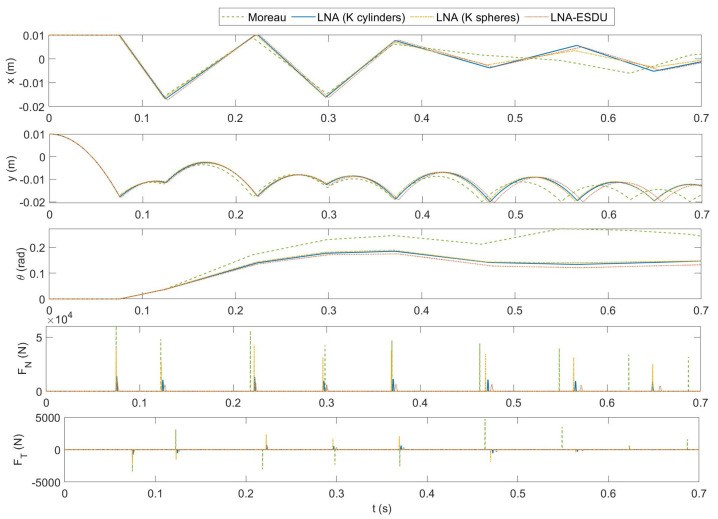
Model comparison considering an asymmetrical impact: from the top to the bottom: *x*-coordinate of $G_{1}$, *y*-coordinate of $G_{1}$, rotation angle θ of body 1, normal contact force $F_{N}$, tangential friction force $F_{T}$.

Observing Figure 8, many of the conclusions of the previous case are also valid in this scenario. The Moreau model is confirmed as the most rigid while the models LNA (K cylinders) and LNA-ESDU are those with less stiffness. While the three continuous models show similar dynamics, the non-smooth Moreau model presents evident differences not only in terms of contact forces but also in terms of gross motion quantities such as position and rotation. This is due to the friction force that amplifies the contact law differences leading to a chaotic behavior that is difficult to predict.

**Figure 9:**
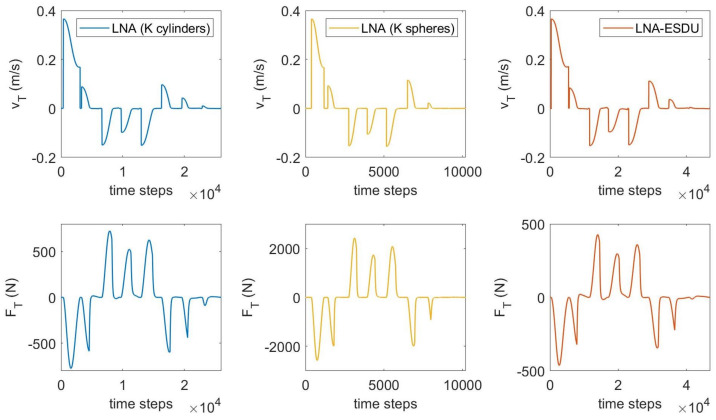
Relative tangential velocity $v_{T}$ and friction force $F_{T}$ in terms of the number of time steps.

To better understand the influence of friction on the dynamics, in Figure 9 the relative tangential velocity $v_{T}$ and the friction force $F_{T}$ are displayed in terms of the time steps for the LNA-based models. Since the number of steps depends on contact duration, stiffer models produce a lower number of iterations and are computationally less expensive. It can be observed that most of the computational time is spent during contact phases since the event-driven scheme reduces the time step, therefore leading to a higher number of steps. Vice-versa, the algorithm increases the time step when no contact is detected. Observing the friction force $F_{T}$, the plots are similar but the peaks grow proportionally to the stiffness of the model. Furthermore, the proposed LNA (*K* cylinders) generates friction forces compared to the LNA-ESDU. This feature is promising as the LNA (*K* cylinders) has lower complexity than LNA-ESDU while providing similar results.

**Figure 10:**
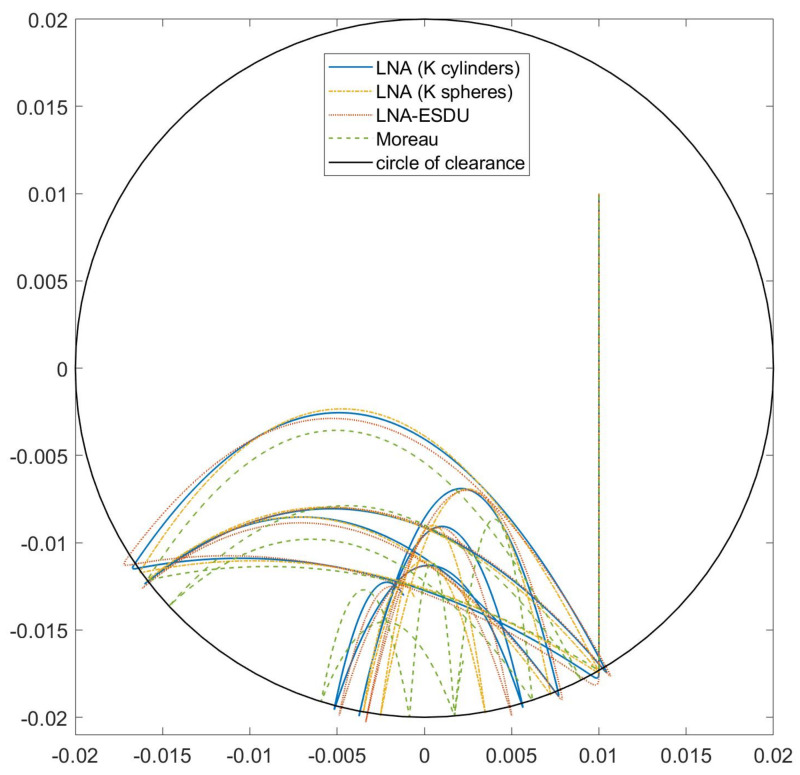
Trajectory of the body–center $G_{1}$ for the four models considering the asymmetrical impact. Starting point at $q_{0}={\left[0.01,0.01,0\right]}^{T}$.

Figure 10 reports the trajectories of the center $G_{1}$ of body 1 for the four models. Due to the presence of contacts, all trajectories are inside the circle of clearance, i.e., a circular region with a radius equal to $\delta =R_{2}-R_{1}$. Points inside this region are subject only to gravity and inertia forces. Points on the boundary or outside are subject also to impact forces.

It can be noted that the less rigid models, namely LNA (*K* cylinders) and LNA-ESDU, have greater impact depths, highlighted by the points outside the circle of clearance. Besides, LNA (K cylinders) confirms to be closer to LNA-ESDU than to LNA (*K* spheres).

### 6.3. CSFH Simulation

**Figure 11:**
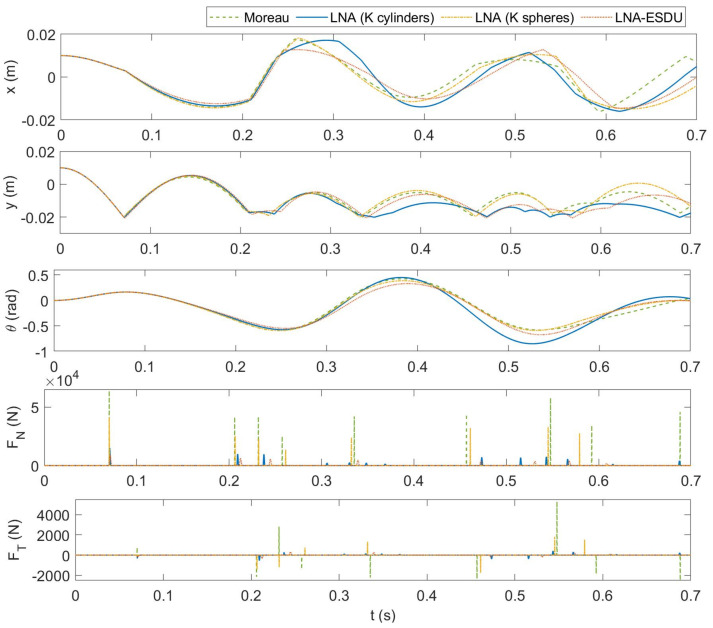
CSFH simulation—comparison of the four models: from the top to the bottom: *x*-coordinate of $G_{1}$, *y*-coordinate of $G_{1}$, rotation angle θ of body 1, normal contact force $F_{N}$, tangential friction force $F_{T}$.

Finally, the complete simulation of a CSFH is presented. Figure 11 reports some relevant results of the numerical simulations. As for the previous case, the models start with similar dynamics from the state $q_{0}={\left[0.01,0.01,0\right]}^{T}$. Comparing the *x*-coordinate of $G_{1}$ in Figure 8 and Figure 11, we can distinguish the influence of the flexure hinge on the horizontal dynamics of the circular flexure pushes the body 1 towards the center $G_{2}$ of body 2. The first impact generates different contact forces and the trajectories rapidly change amplified by the influence of the circular flexure beam. Furthermore, the friction force affects both the translational and the rotational motion. In turn, this modifies the status of the body and therefore the flexure’s response.

**Figure 12:**
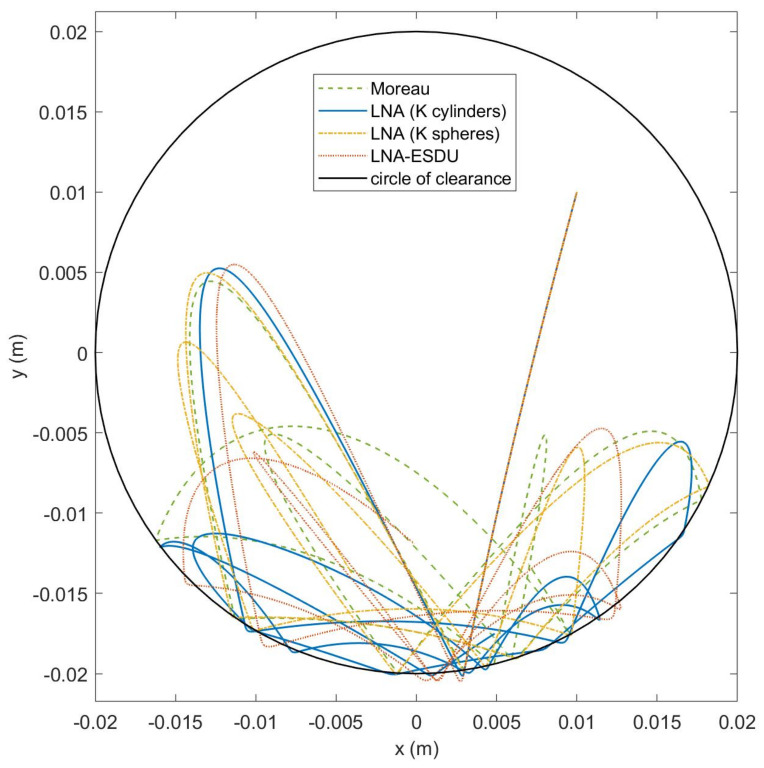
Trajectory of the body–center $G_{1}$ for the four models. Starting point at $q_{0}={\left[0.01,0.01,0\right]}^{T}$.

Finally, Figure 12 reports the trajectories of the center $G_{1}$ of body 1 for the four models. As already pointed out, the elastic force pulls body 1 towards the center deviating the vertical fall that is observed in Figure 10. It can be seen that, from the first impact, the trajectories of the models begin to deviate and the dynamics are chaotic. Even for this case, the less rigid models, namely LNA (K cylinders) and LNA-ESDU, have greater impact depths, highlighted by the points outside the circle of clearance.

## 7. Parametric Analysis

To understand how the parameters of the models influence the CSFH dynamic response we conducted a parametric analysis by varying all the parameters of the model one at a time. The results for the event-driven models are reported in Table 4 where the nominal values are those of Table 1. We have grouped all three event-driven models into a single table because the behavior is similar. The analysis is qualitative and the number of arrows, increasing from one to three, indicates the degree of influence of a parameter on an output variable. Low influence (one arrow) means that the changes in the dynamic response are limited. Medium influence (two arrows) means that the differences gradually amplify as the simulation proceeds. Finally, high influence (three arrows) implies that the differences appear already in the early stages of the simulation generating completely different dynamics.

It can be observed that the radius $R_{1}$, and therefore the radial clearance δ, is a critical parameter for the model. Increasing or decreasing the clearance modifies the kinematics of the impact, anticipating or delaying it, and implies important changes especially on the gross motion (*x*, *y*, θ). Other parameters related to the impact model produce small deviations in the dynamic response. A very different thing happens with regard to the flexure parameters whose modification has strong repercussions on the system dynamics.

Finally, the Table 5 shows the parametric analysis for the time-stepping Moreau’s method. It should be noted that, while following the trend of the event-driven methods, the model is more sensitive to changes in the parameters. Probably, this behavior derives from the higher stiffness of the Moreau’s method.

## 8. Discussion

Comparing the four different models reported in the previous sections revealed important insights. The contact problem with friction was confirmed to be tough. The strong coupling between stiffness and hysteresis loss creates highly non-linear dynamics making the system’s evolution chaotic. This tendency is further amplified by the flexure, being dependent on the position and orientation of the attached bodies. Considering these premises, comparing different impact models would seem useless. Experimentally, it is preferred to quantify the extent of the collision by monitoring, for example, the accelerations produced on bumping bodies. The acceleration is correlated to the impact forces and by observing the first one can quantify the second, which is much more difficult to observe directly. Monitoring the levels of impact forces has important repercussions on various issues of industrial interest such as wear and durability.

To study microcontacts in MEMS application a nanoindenter based experimental setup similar to that proposed in [47] could be designed. A piezoelectric transducer could push the body 1 to touch the body 2. Then, a microprobe, linked to the body 2, could measure forces and displacements.

From the previous numerical results, we can state that the proposed LNA (*K* cylinders) is very close to the experimentally verified LNA-ESDU. Considering the computational efficiency, the Moreau time-stepping method is the fastest. Nevertheless, the impulsive nature of impact forces makes the Moreau model too stiff. The classic LNA method is less stiff than Moreau but it needs more computational resources to resolve the continuous contact. The LNA-ESDU is the most reliable but the slowest method at the same time. Compared to the latter, LNA (*K* cylinders) has the advantage of being simpler by using a constant stiffness instead of a variable one.

The results look promising and worthy of further future developments as the LNA (*K* cylinders) seems to be a good compromise in terms of efficiency and reliability.

## 9. Conclusions

The dynamic model of a CSFH including impacts and friction has been described. First, the flexure hinge elasto-kinematic model has been recalled. Then, the impact kinematics of two conjugate cylindrical surfaces has been formulated and the event-driven contact models have been introduced. These models are based on the detection of the impact instants in correspondence of which a switch among different dynamic models or states is imposed. Based on this class of impact models, we proposed to modify the classic Lankarani-Nikravesh impact model using a generalized stiffness derived from the contact of two cylinders. This solution seemed well-suited to describe the CSFH where two cylindrical conjugate surfaces collide. The proposed model has been equipped with the modified Ambrósio friction model. Then, the non-smooth Moreau’s scheme has been recalled. The latter is a time-stepping method that does not require the detection of impact points and the consequent change of state.

In the numerical part, the proposed method, the Moreau scheme, and two continuous event-driven schemes including the classic Lankarani-Nikravesh-Ambrósio model and its evolution obtained by the ESDU constitutive law have been compared in different impact scenarios. All numerical simulations revealed that the dynamics are strongly influenced by the impact formulation and its parameters. Furthermore, due to the high non-linearity of the problem, the differences are further amplified by the presence of the flexure. The proposed method not only provided indentation, impact, and friction force values comparable to those provided by the experimentally verified LNA-ESDU model but has the advantage of requiring lower computational resources equal to those needed by the classic LNA method.

The parametric analysis revealed that some parameters such as the radial clearance or flexure parameters have a strong influence on system dynamics. This can be very useful in helping designers to establish the right manufacturing processes and dimensional tolerances.

The present investigation is expected to be a first step towards the understanding of the dynamic behavior of CSFH. Furthermore, the benefits introduced by the method could be important in developing control strategies. For example, the impact model could characterize the displacements of a micro-gripper equipped with CSFHs in function of the comb-drives actuation voltage. In this way, it would be possible to fully exploit the potential of the CSFH by taking into account the contact dynamics among the conjugated surfaces and ensuring, at the same time, control over the maximum stresses that the materials can withstand during the impact phase.

## Figures and Tables

**Table 1:** Common geometric, inertial, and structural parameters of the CSFH used in the numerical simulations.

Notation	Description	Value	Unit
β	hinge opening angle	300	(${}^{\circ }$)
ρ	hinge radius	0.144	(m)
$\vec{OF}$	hinge center coordinates	${\left[0.22,0.1\right]}^{T}$	(m)
$\vec{G_{1}C_{1}}$	hinge attachment point to body 1	${\left[0.2920,-0.02471\right]}^{{\left(1\right)}^{T}}$	(m)
$\vec{OC_{2}}$	hinge attachment point to body 2	${\left[0.364,0.1\right]}^{T}$	(m)
*h*	cross-section height of the hinge	0.005	(m)
*b*	cross-section width of the hinge	0.025	(m)
$R_{1}$	body 1 radius	0.18	(m)
$R_{2}$	body 2 radius	0.20	(m)
$\nu_{h}$	hinge Poisson’s ratio	0.3	(-)
$E_{h}$	hinge Young’s modulus	100	(GPa)
$m_{1}$	body 1 mass	10	(kg)
$I_{1}$	body 1 moment of inertia	0.1617	(kg m^2^)
$\nu_{1},\nu_{2}$	body 1, 2 Poisson’s ratio	0.3	(-)
$E_{1},E_{2}$	body 1, 2 Young’s modulus	100	(GPa)
$f_{a}$	adherence coefficient	0.11	(-)
$f_{d}$	dynamic friction coefficient	0.055	(-)
$c_{r}$	restitution coefficient	0.9	(-)

**Table 2:** Lankarani-Nikravesh impact model [20].

Notation	Description	Value	Unit
*K* (spheres)	generalized stiffness parameter for Equation (14)	47.35	(GPa)
*K* (cylinders)	generalized stiffness parameter for Equation (16)	2.27	(GPa)
*n*	nonlinear exponent factor	1.5	(-)

**Table 3:** Modified Ambrósio friction model [41].

Notation	Description	Value	Unit
$v_{0}$	lower tolerance for the tangential velocity	1 × 10${}^{-4}$	(m/s)
$v_{1}$	upper tolerance for the tangential velocity	1 × 10${}^{-2}$	(m/s)

**Table 4:** Parametric analysis for the event-driven models. One arrow (low influence), two arrows (medium influence), three arrows (high influence).

	*x*	*y*	θ	$F_{N}$	$F_{T}$
$R_{1}\pm 1$ (mm)	↑↑↑	↑↑↑	↑↑↑	↑↑	↑
$m_{1}\pm 0.1$ (kg)	↑	↑	↑	↑	↑
$I_{1}\pm 0.001$ (kg m^2^)	↑	↑	↑	↑	↑
$E_{1,2}\pm 10$ (GPa)	↑	↑	↑	↑	↑
$c_{r}\pm 0.01$ (-)	↑	↑	↑	↑	↑
$f_{d}\pm 0.005$ (-)	↑	↑	↑	↑	↑
ρ±1 (mm)	↑↑	↑↑	↑	↑↑	↑↑
b±1 (-)	↑	↑	↑	↑	↑
h±1 (mm)	↑↑	↑↑	↑↑	↑↑	↑↑
β±1 (${}^{\circ }$)	↑↑↑	↑↑↑	↑↑	↑↑↑	↑↑
$E_{h}\pm 10$ (GPa)	↑↑↑	↑↑↑	↑↑↑	↑↑	↑↑

**Table 5:** Parametric analysis for the time-stepping Moreau’s method. One arrow (low influence), two arrows (medium influence), three arrows (high influence).

	*x*	*y*	θ	$F_{N}$	$F_{T}$
$R_{1}\pm 1$ (mm)	↑↑↑	↑↑↑	↑↑↑	↑↑	↑↑
$m_{1}\pm 0.1$ (kg)	↑↑	↑↑	↑↑	↑↑	↑↑
$I_{1}\pm 0.001$ (kg m^2^)	↑	↑	↑	↑	↑
$c_{r}\pm 0.01$ (-)	↑↑	↑↑	↑↑	↑↑	↑↑
$f_{d}\pm 0.005$ (-)	↑↑	↑↑	↑	↑	↑
$f_{a}\pm 0.01$ (-)	↑	↑	↑	↑	↑
ρ±1 (mm)	↑↑	↑↑	↑	↑↑	↑↑
b±1 (-)	↑	↑	↑	↑	↑
h±1 (mm)	↑↑↑	↑↑↑	↑↑↑	↑↑	↑↑
β±1 (${}^{\circ }$)	↑↑↑	↑↑↑	↑↑	↑↑	↑↑
$E_{h}\pm 10$ (GPa)	↑↑↑	↑↑↑	↑↑↑	↑↑↑	↑↑↑
